# Immune complexes shift the TLR-induced cytokine production of distinct polarized human macrophage subsets towards IL-10

**DOI:** 10.1186/1479-5876-9-S2-P1

**Published:** 2011-11-23

**Authors:** Carmen Ambarus, Lenny Geurts-van Bon, Mark Wenink, Paul Peter Tak, Timothy Radstake, Dominique Baeten

**Affiliations:** 1 Dept. of Clinical Immunology and Rheumatology, Academic Medical Center/University of Amsterdam, The Netherlands; 2 Dept. of Rheumatology, Radboud University Nijmegen Medical Center Nijmegen and Nijmegen Institute for Infection, Inflammation and Immunity, Nijmegen, The Netherlands

## Background

Costimulation of macrophages with immune complexes (ICs) and TLR ligands leads to alternative activation of murine macrophages. Most studies on human monocytes, dendritic cells and M-CSF differentiated macrophages indicate that ICs induce an increased TNF production. This study aimed to clarify the effect of ICs on the pro- versus anti-inflammatory profile of human macrophages.

## Materials and methods

Monocytes isolated from peripheral blood of healthy donors were polarized for 4 days by IFN-γ, IL-4, IL-10, GM-CSF, M-CSF, or LPS, in the presence or absence of soluble heat aggregated gamma-globulins (HAGGs). The expression of previously validated phenotypic polarization markers was assessed by flow cytometry. To assess the effect of ICs on cytokine production, polarized macrophages were stimulated with HAGGs alone or in combination with three TLR ligands for 20 hours. TNF, IL-6, and IL-10 levels in the supernatants were measured by ELISA. IL-23p19, IL-12p35, and IL-10 mRNA levels were measured by RT-qPCR after 7 hours of stimulation.

## Results

ICs alone or in combination with the other polarizing stimuli did not have any significant effect on the expression of polarization markers. TLR stimulation induced a high TNF and IL-6 production by IFN-γ polarized macrophages, while IL-10 production was similar in IFN-γ, IL-4, and IL-10 polarized macrophages. Stimulation with ICs alone did not modulate the cytokine production. However, ICs enhanced the TLR-induced IL-10 production in all macrophage subsets, excepting IL-4 polarized macrophages. TLR-mediated TNF and IL-6 production was differentially modulated by ICs for each macrophage subset. The increased IL-10 expression was confirmed at mRNA level, while IL-23p19 and IL-12p35 expression was not affected by incubation with ICs.

## Conclusion

Soluble ICs alone did not alter the phenotype and cytokine production of in vitro polarized human macrophages. In combination with TLR-ligands, however, ICs shifted the cytokine profile of distinct human macrophage subsets towards IL-10 production.

